# In vitro study on effect of bardoxolone methyl on cisplatin-induced cellular senescence in human proximal tubular cells

**DOI:** 10.1007/s11010-021-04295-y

**Published:** 2022-01-01

**Authors:** Yoshifumi Kurosaki, Akemi Imoto, Fumitaka Kawakami, Motoshi Ouchi, Asuka Morita, Masanori Yokoba, Tsuneo Takenaka, Takafumi Ichikawa, Masato Katagiri, Rikke Nielsen, Naohito Ishii

**Affiliations:** 1 Department of Molecular Medical Biology, Kitasato University Graduate School of Medical Sciences, Kitasato 1-15-1, Minami-ku, Sagamihara-shi, Kanagawa 252-0373 Japan; 2 Department of Pharmacology and Toxicology, Dokkyo Medical University School of Medicine, Tochigi, Japan; 3 Department of Clinical Medicine, Kitasato University Graduate School of Medical Sciences, Kanagawa, Japan; 4 Department of Nephrology, International University of Health and Welfare Graduate School of Medicine, Tokyo, Japan; 5 Department of Biomedicine, Aarhus University, Århus, Denmark

**Keywords:** CDDO-Me, Cellular senescence, Acute Kidney injury, Nrf2, Apoptosis

## Abstract

Bardoxolone methyl [methyl-2-cyano-3, 12-dioxooleana-1, 9(11)dien-28-oate (CDDO-Me)], an activator of the nuclear factor erythroid-derived 2-related factor2 pathway, is a potential therapeutic candidate for the treatment of kidney diseases. However, its effect against cellular senescence remains unclear. This study aimed to investigate whether CDDO-Me protects cells against cisplatin-induced cellular senescence using an in vitro model. The human renal proximal tubular epithelial cell line HK-2 was treated with cisplatin for 6 h, followed by treatment with or without CDDO-Me (0.1 or 0.2 μmol/L). Senescence markers were analyzed using western blotting and real-time PCR. Apoptosis was evaluated through TUNEL staining. Cisplatin induced changes in the levels of markers specific for proliferation, cell cycle, and senescence in a time- and dose-dependent manner. Furthermore, IL-6 and IL-8 levels in the culture medium increased markedly. These data suggested that cellular senescence-like alterations occurred in HK-2 cells exposed to cisplatin. CDDO-Me treatment reversed the cisplatin-mediated alterations in the levels of cellular senescence markers. The antioxidant enzymes, *HO1*, *NQO1*, *GPX1*, and *CAT* were upregulated by CDDO-Me treatment. Furthermore, CDDO-Me treatment induced apoptosis in cisplatin-exposed HK-2 cells. Pretreatment with Ac-DEVD-CHO, the caspase inhibitor, suppressed the reversal effect of CDDO-Me against cisplatin-induced cellular senescence-like alterations. This study showed that CDDO-Me attenuated cisplatin-induced premature senescence of HK-2 cells. This beneficial effect may be related to Nrf2 activation. Our findings also showed that CDDO-Me induced apoptosis in cisplatin-treated HK-2 cells, potentially protecting the kidneys from cellular senescence. CDDO-Me appears to be a candidate treatment for acute kidney injury.

**Supplementary Information:**

The online version contains supplementary material available at 10.1007/s11010-021-04295-y.

## Introduction

Acute kidney injury (AKI) is a complex syndrome characterized by renal dysfunction and is associated with high morbidity and mortality worldwide. Patients with post-AKI are at an increased risk for chronic kidney disease, CKD [1–3]. Proximal tubule injury caused by AKI triggers several features of CKD by inducing inflammation and interstitial fibrosis [4]. Therefore, it is important to protect the proximal tubules for preventing the progression of AKI to CKD; however, the definitive evidence for this effect is lacking.

There is an association between cellular senescence in proximal tubular epithelial cells (PTCs) and CKD in the animal models of hypertension [5] or diabetes [6]. Senescence is a tumor suppressor mechanism by which cells adapt to DNA damage, oxidative stress, and telomere shortening. Senescence induces cell cycle arrest in cells exposed to various stresses [7, 8]. Senescent cells secrete pro-inflammatory molecules in order to recruit immune cells. This behavior is termed as senescence-associated secretory phenotype, SASP [9]. These pro-inflammatory molecules, including cytokines and chemokines, can lead to the clearance of senescent cells in the damaged tissue [10, 11]. In contrast, the chronic secretion of these SASP factors promotes inflammation and epithelial-to-mesenchymal transition, thereby reducing the functional reserve of tissues. Thus, senescence is protective in the acute phase; however, chronic senescence can be harmful to tissue homeostasis. Senescent cells undergo an anti-apoptotic state [12–14], leading to the constitutive secretion of SASP factors. Chronic secretion of SASP factors can spread senescence to nearby cells [15]; therefore, the removal of senescent cells by activating apoptosis seems to be a critical mechanism for protecting the proximal tubule and preventing CKD progression. In fact, much effort has been made to eliminate senescent cells using novel drugs called senolytics. These drugs remove senescent cells by inducing apoptosis [16] or by blocking their resistance to apoptosis [17, 18]. Therefore, protecting PTCs from cellular senescence could prevent CKD development; however, effective treatments that target senescence are lacking.

Methyl-2-cyano-3, 12-dioxooleana-1, 9(11)dien-28-oate (CDDO-Me), also known as bardoxolone methyl, is a semi-synthetic triterpenoid and the most potent activator of the nuclear factor erythroid-derived 2-related factor2 (Nrf2) pathway [19–21]. Besides activating Nrf2, CDDO-Me upregulates the antioxidant response and suppresses pro-inflammatory signaling reducing oxidative stress and inflammation, and promoting mitochondrial function [22, 23]. CDDO-Me and its analogs have beneficial effects on CKD associated with type 2 diabetes [24, 25], obesity [26], and angiotensin-induced kidney injury [27]. Furthermore, CDDO-Me induces apoptosis [28]; therefore, CDDO-Me could be beneficial for eliminating cellular senescence. CDDO-Me is an attractive therapeutic candidate for managing cellular senescence in kidney diseases, but the precise effects remain unknown.

Cisplatin is one of the most widely used anti-cancer drugs; however, its use is limited because of its nephrotoxicity, which causes AKI [29]. Cisplatin treatment is widely used as a model for AKI; it induces cellular senescence, both in vitro [30, 31] and in vivo [32, 33]. The aim of this study was, therefore, to investigate whether CDDO-Me protects PTCs against cisplatin-induced cellular senescence to explain the beneficial effect of CDDO-Me.

## Materials and methods

### Cell culture

The human renal proximal tubular epithelial cell line HK-2 was cultured in Dulbecco’s Modified Eagle’s Medium: Nutrient Mixture F-12 (DMEM/F12, Fujifilm Wako Chemical Co., Osaka, Japan) supplemented with 10% fetal bovine serum (FBS, Biosera, Inc., Nuaille, France) and 1% penicillin–streptomycin, as previously described [34]. Cells were treated with 0–50 µmol/L cisplatin in low-glucose DMEM containing 0.1 mg/mL human serum albumin for 6 h. The medium was then replaced with fresh medium with or without 0.1–0.2 μmol/L CDDO-Me (Sigma-Aldrich Co., St. Louis, MO, USA). The cells were treated with 5 or 50 µmol/L of the caspase inhibitor, Ac-DEVD-CHO (Selleckchem, Houston, TX, USA), for 60 min before adding CDDO-Me to the medium. Cells were cultured for 24–72 h, and proteins or mRNA were extracted at the indicated points.

### Western blot (WB) analysis

The cultured cells were solubilized in lysis buffer (150 mmol/L NaCl, 50 mmol/L Tris–HCl, 5 mmol/L EDTA–2Na, 1% Triton X-100, and 1 tablet/10 mL complete mini EDTA-free) and centrifuged at 15,000 × *g* at 4 °C for 30 min. Samples were separated by SDS-PAGE and then transferred to PVDF membranes. The membranes were first blocked in a buffer containing 25 mmol/L Tris–HCl (pH 7.4), 150 mmol/L NaCl, 0.1% Tween 20, and 4% skim milk for 1 h and then incubated with primary antibodies at 4 °C overnight. This was followed by incubation with horseradish peroxidase-conjugated secondary antibodies for 1 h. Primary antibodies against human p21^Waf1/Cip1^, p16^INK4a^, phosphorylated H2AX (Ser139, γ-H2AX), retinoblastoma (Rb), phosphorylated Rb (Ser780, pRb), cyclin D, and caspase-3 were all purchased from Cell Signaling Technology, Inc. (Beverly, MA, USA). Antibodies against human cyclin A (Novocastra Laboratories Ltd., Newcastle, UK), p16^INK4a^ (BD Biosciences, Inc., Farmingdale, NY, USA), Ki-67 (Dako from Agilent, Santa Clara, CA, USA), p62/SQSTM1, and β-actin (Santa Cruz Biotechnology, Santa Cruz, CA, USA) were also used. The immunoreactive proteins were then detected by enhanced chemiluminescence (GE Healthcare, Fairfield, CT, USA). Immunoblots were quantified using the CS Analyzer 3.0 software (ATTO, Tokyo); β-actin expression was used as the internal control.

### Real-time reverse transcription-PCR (RT-qPCR)

Total RNA and cDNA from HK-2 cells were prepared using the ISOGEN-II (Nippon Gene, Tokyo) and Prime Script RT-PCR kits (Takara Bio, Shiga), according to the manufacturer’s instructions. The primers used for qPCR are shown in Supplementary Table 1. qPCR was performed on a 7500 Real-Time PCR System (Applied Biosystems) in a 96-well reaction plate using Power SYBR Green. The mRNA expression of the target genes was normalized to that of *GAPDH*, using the delta–delta *C*_t_ method.

### Cell cycle analysis using flow cytometry

HK-2 cells were harvested, washed, and resuspended in phosphate buffered saline. Cells were then fixed with 70% ethanol and stored at 4 °C overnight. Subsequently, cells were incubated with 100 µg/mL RNase for 30 min at 37 °C and stained with 5 µg/mL propidium iodide (PI) for 10 min. Flow cytometry analyses were performed using a BD FACSCalibur (BD Biosciences). Cell cycle phase distributions were determined using Modfit LT software version 3.0 (Verity Software House, Topsham, ME, USA).

### Determination of cytokine levels by ELISA

Following exposure to cisplatin, supernatants were collected from the culture medium. Cytokine levels were determined using ELISA kits for IL-6 and IL-8 (R&D Systems, Minneapolis, MN, USA) as per the manufacturer’s instructions.

### TdT-mediated dUTP-biotin nick end labeling (TUNEL) staining

TUNEL staining was performed using a commercial kit (Takara Bio), following each treatment described above. The percentage of apoptotic cells was determined by counting the TUNEL-positive cells and the total number of cells (nucleus) in four to six photomicrographs (× 200 magnification, approximately 1500 cells) by a blinded observer. This experiment was performed thrice, on different days. The apoptosis rate was expressed as the means ± standard deviation (SD) from three independent experiments.

### Statistical analysis

All data are reported as the mean ± SD. The RT-qPCR and WB analysis data are presented as the fold change relative to the controls (untreated cells). The RT-qPCR data were evaluated using Students *t*-test to assess differences between the control and cisplatin-treated cells. Other data were analyzed using analysis of variance (ANOVA) with post hoc comparisons using the Student–Newman–Keuls method. *P* values < 0.05 were considered statistically significant.

## Results

### Cisplatin treatment induces cellular senescence-like alterations in HK-2 cells

**Fig. 1:**
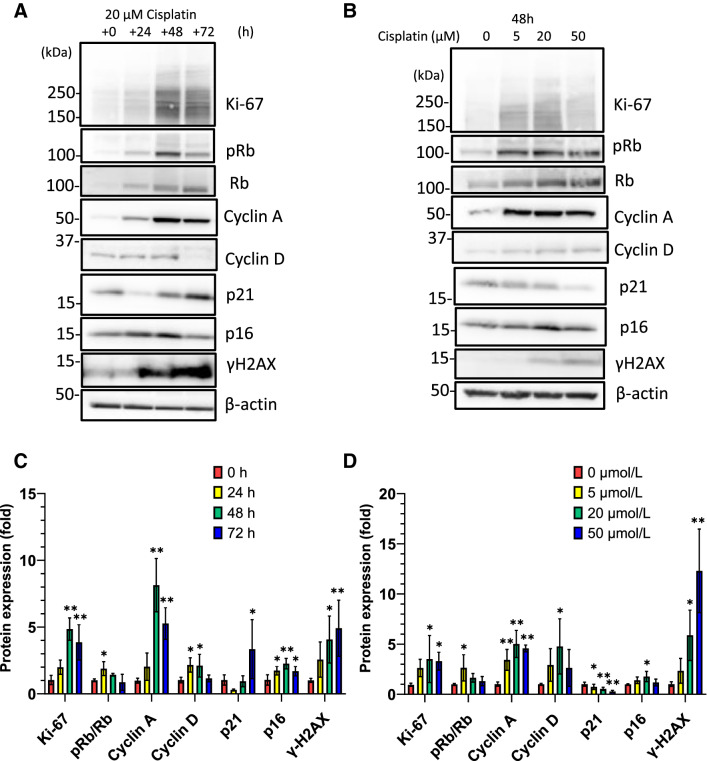
Cisplatin induces cellular senescence-like alterations in HK-2 cells. HK-2 cells were treated with 0–50 µmol/L cisplatin, and western blot analysis revealed changes in the expression levels of proliferation, cell cycle, and senescence markers in a time- (**A**) and dose- (**B**) dependent manner. Densitometric analysis is shown in **C**–**D**. *n* = 4–6, *P < 0.05, **P < 0.01

**Fig. 2:**
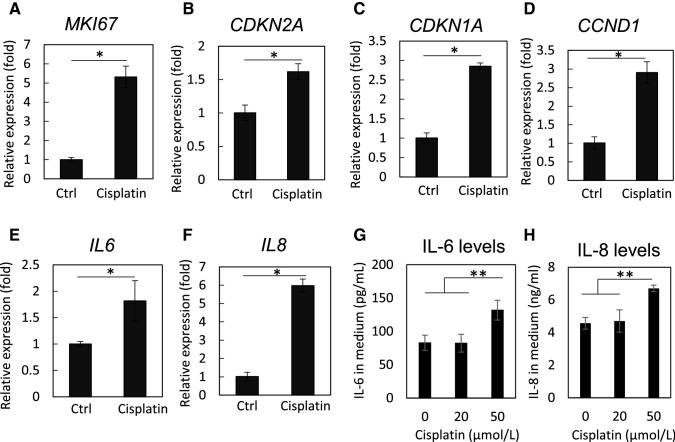
Cisplatin treatment induces the increase in senescence-related gene transcriptions and cytokine secretion. The mRNA expression of senescence-related genes, *MKI67* (**A**), *CDKN2A* (**B**), *CDKN1A* (**C**), *CCND1* (**D**), *IL-6* (**E**), and *IL-8* (**F**), after cisplatin (20 µmol/L) treatment was determined by qPCR. Cytokine levels, IL-6 (**G**) and IL-8 (**H**), in cultured medium of cisplatin (20 or 50 µmol/L)-exposed cells were quantified by ELISA. *n* = 3, *P < 0.05, **P < 0.01

First, we examined whether cisplatin exposure induces cellular senescence in HK-2 cells. Cisplatin treatment induced alterations in the expression of proliferation, cell cycle, and senescence markers in a time- and dose-dependent manner (Fig. 1). The expression of Ki-67, a proliferation marker, significantly increased after exposure to 20 μmol/L cisplatin; it decreased after exposure to 50 μmol/L cisplatin compared to that after exposure to 20 μmol/L cisplatin. The expression of the cell cycle markers pRb, Rb, cyclin D, and cyclin A increased in cisplatin-exposed HK-2 cells. Cisplatin treatment increased the levels of p16^INK4a^ in a time-dependent manner. The expression level of p21^Waf1/Cip1^ was decreased at 48 h; however, it was increased at 72 h. Cisplatin treatment increased the level of γ-H2AX, a DNA damage marker. The mRNA expression of *MKI67* (encoding Ki-67), *CDKN1A* (encoding p21^Waf1/Cip1^), *CDKN2A* (encoding p16^INK4a^), and *CCND1* (encoding cyclin D1) increased 48 h after exposure to 20 μmol/L cisplatin (Fig. 2A–D). Furthermore, the mRNA levels of *IL-6* and *IL-8* increased markedly (Fig. 2E, F). An increase in the cytokine levels was observed in the culture medium of cells exposed to 50 μmol/L cisplatin (Fig. 2G, H). These data suggest that cisplatin treatment induced proliferation, DNA damage, and subsequent cellular senescence-like alterations in HK-2 cells.

### CDDO-Me reverses cisplatin-induced cellular senescence-like alterations in HK-2 cells

**Fig. 3:**
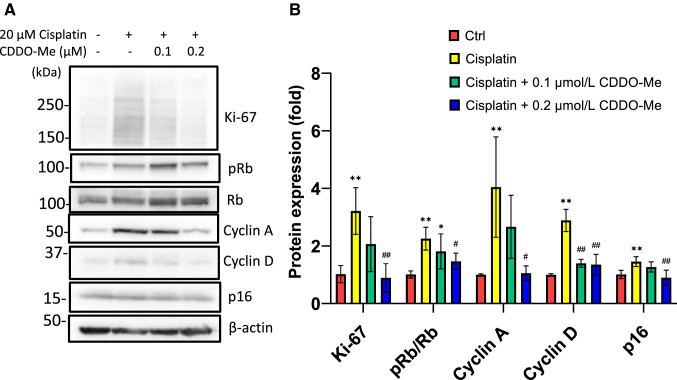
CDDO-Me treatment reversed the cisplatin-induced cellular senescence phenotype in HK-2 cells. HK-2 cells were exposed to 20 µmol/L cisplatin for 6 h, followed by treatment with or without CDDO-Me (0.1 or 0.2 µmol/L). Proteins were collected 48 h after treatment and analyzed by western blotting (**A**), and protein bands were quantified by densitometry (**B**). *n* = 4–6, *P < 0.05, **P < 0.01 vs. control (Ctrl); ^+^*P* < 0.05, ^++^*P* < 0.01 vs. cisplatin

**Fig. 4:**
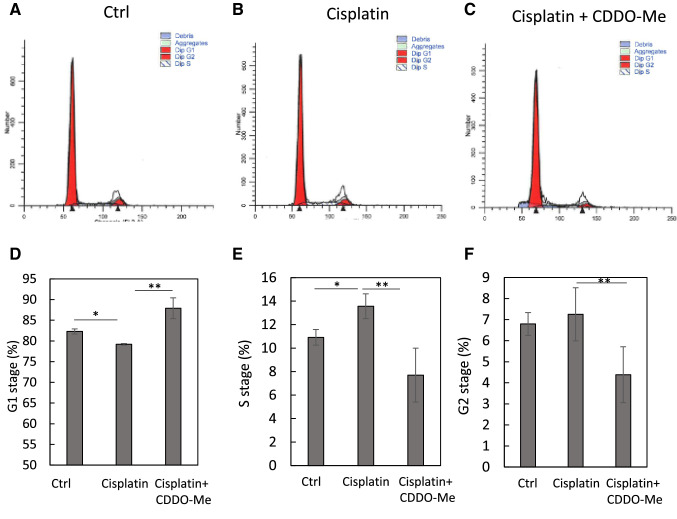
CDDO-Me protects cells against cisplatin-induced abnormalities in cell cycle. Cisplatin (20 µmol/L)-exposed cells were treated with 0.2 µmol/L CDDO-Me for 48 h. Cells were harvested and stained with propidium iodide, and then analyzed by flow cytometry (**A**–**C**). The statistics of each cell cycle phase (**D**–**F**). *n* = 3, *P < 0.05, **P < 0.01

CDDO-Me treatment normalized the cisplatin-induced alterations in the expression of the cellular senescence markers Ki-67, cyclin A, cyclin D, pRb/Rb, and p16^INK4a^ in HK-2 cells (Fig. 3). Cell cycle analysis using cytometry revealed that CDDO-Me treatment ameliorated cisplatin-induced cell cycle abnormalities (Fig. 4). Cisplatin markedly decreased the percentage of cells in the G1 phase and increased that in the S phase. The cells in the G2 phase also tended to increase after cisplatin treatment. However, CDDO-Me treatment increased the number of G1-phase cells and decreased that of S- and G2-phase cells.

### CDDO-Me upregulates the expression of antioxidant enzymes

**Fig. 5:**
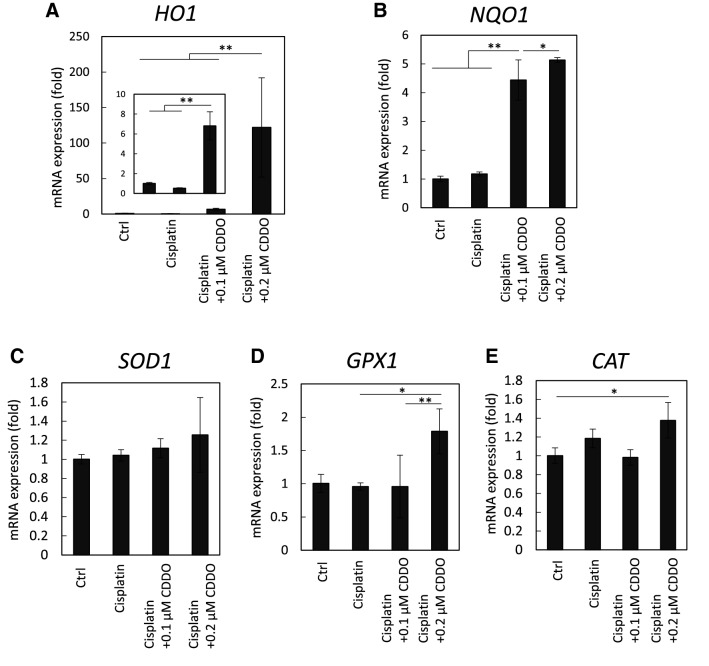
CDDO-Me induces ARE-related gene transcription by activating the Keap1-Nrf2 pathway. HK-2 cells were exposed to 20 µmol/L cisplatin for 6 h, followed by treatment with or without CDDO-Me (0.1 or 0.2 µmol/L). The mRNA expression levels of Nrf2-related genes were analyzed by qPCR 48 h after treatment: heme oxygenase-1 (**A**), NAD(P)H:quinone oxidoreductase (**B**), superoxide dismutase 1 (**C**), glutathione peroxidase 1 (**D**), and catalase (**E**). *n* = 3, *P < 0.05, **P < 0.01

To evaluate the mechanisms by which CDDO-Me protected the cisplatin-exposed HK-2 cells against cellular senescence-like alterations, we evaluated whether the expression of antioxidant enzyme gene were upregulated under the present conditions (Fig. 5). The mRNA of antioxidant enzymes, heme oxygenase-1 (*HO1*), NAD(P)H:quinone oxidoreductase (*NQO1*), glutathione peroxidase 1 (*GPX1*), and catalase (*CAT*), were upregulated by CDDO-Me treatment. However, CDDO-Me treatment did not significantly increase the mRNA expression of superoxide dismutase 1 (*SOD1*).

### CDDO-Me induces apoptosis in cisplatin-treated HK-2 cells

**Fig. 6:**
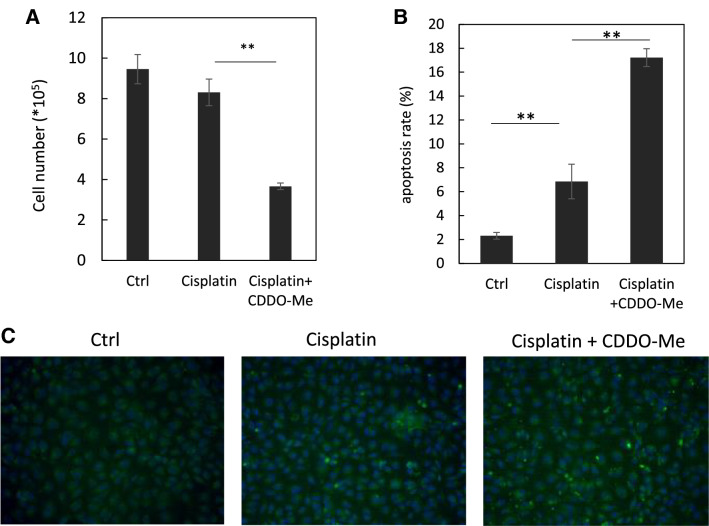
CDDO-Me treatment decreases the cell number in cisplatin-exposed HK-2 cells by inducing apoptosis. Cisplatin (20 µmol/L)-exposed HK-2 cells were cultured in medium with or without 0.2 µmol/L CDDO-Me. Cell numbers were counted at 48 h (**A**). *n* = 3, **P < 0.01. Apoptotic cells were detected in cisplatin (20 µmol/L)-exposed cultures after treatment with or without 0.2 µmol/L CDDO-Me by TUNEL staining (original magnification × 200) (**B**), and the number of apoptotic cells was expressed as the apoptosis rate (**C**). Data are presented as mean ± SD from three independent experiments. **P < 0.01

**Fig. 7:**
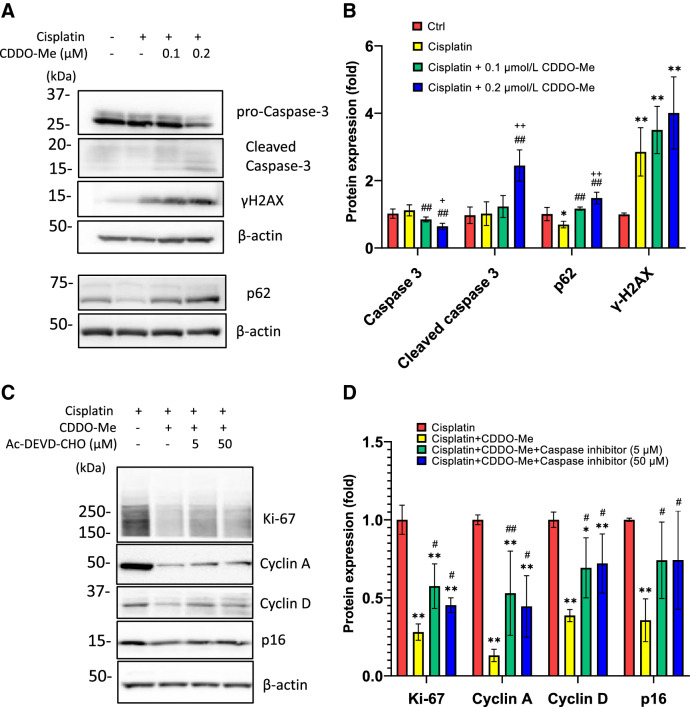
CDDO-Me induces apoptosis in cisplatin-treated HK-2 cells. HK-2 cells were exposed to 20 µmol/L cisplatin for 6 h, followed by treatment with or without CDDO-Me (0.1 or 0.2 µmol/L). Apoptotic marker proteins were analyzed by western blotting (**A**), and protein bands were quantified by densitometry (**B**). *n* = 4–6, *P < 0.05, **P < 0.01 vs. control (Ctrl), ^#^*P* < 0.05, ^##^*P* < 0.01 vs. cisplatin, ^+^*P* < 0.05, ^++^*P* < 0.01 vs. cisplatin + CDDO-Me (0.1 µmol/L). Cisplatin-exposed cells were treated with 5 or 50 µmol/L Ac-DEVD-CHO, a caspase inhibitor, for 60 min prior to adding 0.2 µmol/L CDDO-Me to the medium. Senescence markers were analyzed using western blotting (**C**) and the expression was quantified using densitometry (**D**). n = 4–5, *P < 0.05, **P < 0.01 vs. cisplatin, ^#^*P* < 0.05, ^##^*P* < 0.01 vs. cisplatin + CDDO-Me

The effect of CDDO-Me on apoptosis was evaluated because CDDO-Me treatment significantly decreased the cell number in cisplatin-exposed HK-2 cells (Fig. 6A). CDDO-Me induced apoptosis in cisplatin-exposed HK-2 cells; however, only cisplatin treatment also induced apoptosis, as observed using TUNEL staining (Fig. 6B, C). WB analysis confirmed that CDDO-Me treatment induced apoptosis, decreased the level of pro-caspase-3, and increased the level of cleaved caspase-3 and p62/SQSTM1 in HK-2 cells (Fig. 7A, B). CDDO-Me accelerated the phosphorylation of H2AX in cisplatin-treated HK-2 cells. To elucidate whether CDDO-Me reverses cisplatin-induced cellular senescence-like alterations via apoptosis, cisplatin-exposed cells were treated with Ac-DEVD-CHO, a caspase inhibitor, before the CDDO-Me treatment. Ac-DEVD-CHO treatment inhibited the decrease in Ki-67, cyclin A, cyclin D, and p16^INK4a^ expression levels induced by CDDO-Me (Fig. 7C, D).

## Discussion

CDDO-Me therapy improves renal function in CKD patients, in clinical trials [24, 25, 35]. However, the renal protective mechanism of CDDO-Me on the AKI-CKD transition remains unknown. Protecting the proximal tubules from AKI, which causes cellular senescence, is important for preventing CKD development. This study elucidated the protective effect of CDDO-Me on cisplatin-induced cellular senescence in cultured human PTCs, HK-2, which is one of the most used cells for the study of senescent PTCs [36–38]. In this study, WB analysis revealed an increased expression of Ki-67 in HK-2 cells following cisplatin treatment; chronic increase in proliferation induces cellular senescence in PTCs [39]. Accelerated proliferation may be one mechanism by which cellular senescence is induced in cisplatin-treated cells. In addition to a pool of proliferating cells, elevated cell cycle arrest markers were observed, indicating the presence of a pool of senescent cells following cisplatin treatment. This was demonstrated by the upregulation of cyclin-dependent kinase inhibitors, such as p16^INK4a^, p21^Waf1/Cip1^, and phosphorylated Rb in cells exposed to cisplatin for 72 h. An increase in p21^Waf1/Cip1^ and p16^INK4a^ induces G2/M arrest through the phosphorylation of Rb [40]. Flow cytometric analysis showed an increase in the number of S-phase cells in cisplatin-treated cultures. Thus, cisplatin treatment seemed to be associated with S- or G2/M-phase arrest by regulating p16^INK4a^ and/or p21^Waf1/Cip1^. Cisplatin stalls cell proliferation by damaging DNA and inhibiting DNA synthesis, which is cytotoxic during the S phase [41]. In fact, γ-H2AX, a DNA damage marker, was elevated in cisplatin-treated cells. Furthermore, cisplatin treatment induced the upregulation and secretion of IL-6 and IL-8 in the medium. These results indicate that cisplatin-exposed cells undergo premature cellular senescence, consistent with the results from previous studies [30, 31].

This study showed that CDDO-Me treatment decreased senescence markers in PTCs exposed to cisplatin. Upon activation by CDDO-Me, Nrf2 translocates to the nucleus and induces the transcription of antioxidant enzyme genes via the antioxidant response element, ARE [42]. This was demonstrated by the upregulation of the antioxidant enzymes following the CDDO-Me treatment of the cisplatin-treated HK-2 cells. Aleksunes et al. elucidated using in vivo studies that cisplatin accelerates cell proliferation and secretion of IL-1β, IL-6, and TNF-α in the kidney; importantly, this phenotype was inhibited by CDDO-lm, an analog of CDDO-Me, through the increase of Nrf2 signaling [43]. Treatment with the antioxidant N-acetylcysteine ameliorates cellular senescence in PTCs induced by multiple cisplatin treatments [32]. Furthermore, an in vitro study using human PTCs showed that the upregulation of the antioxidant genes by Nrf2 enhanced cell viability [44]. These studies suggest that removing oxidative stress via the Keap1-Nrf2 pathway protects PTCs from cellular senescence.

Overdosing with CDDO-Me induces apoptosis [28]. Eliminating the senescent cells by activating apoptosis has a protective effect on tissue aging [16–18]. Therefore, we evaluated whether CDDO-Me induced apoptosis and reversed senescence via an apoptotic mechanism. We showed that CDDOM-Me decreased the cell number, which occurs through the apoptotic mechanism. CDDO-Me treatment increased the level of cleaved caspase-3 and the number of TUNEL-positive cells. Furthermore, CDDO-Me accelerated the phosphorylation of H2AX, which occurs in response to DNA damage and induces apoptosis by mitochondrial cytochrome C release [45]. Additionally, CDDO-Me increases p62/SQSTM1 expression, which plays a critical role in both autophagy and apoptosis [46, 47]. Inhibition of apoptosis suppressed the reversal effect of CDDO-Me against cisplatin-induced cellular senescence-like alterations. Therefore, CDDO-Me induces apoptosis and suppressed cellular senescence in cisplatin-treated cells. The activation of apoptosis could contribute to the removal of senescent cells and improved kidney functions.

The use of only one cell line was a limitation of this study. HK-2 cells were used in this study, because senescence could be easily induced by treating the HK-2 cells with d-serine, indoxyl sulfate, and hydrogen peroxide [36–38]. Additionally, HK-2 cells maintain biochemical properties similar to that in the in vivo proximal tubule cells [48, 49]. Therefore, this model was well-suited for this study. However, HK-2 cells lack expression of organic anion transporter 1, 3, and organic cation transporter 2, which are important for drug metabolism [50]. Therefore, drug metabolism in HK-2 might not be completely similar to that in the in vivo state. It is unclear whether CDDO-Me removes senescent cells selectively in vivo; therefore, we cannot conclude that activation of apoptosis by CDDO-Me protects against kidney injury in humans. Further in vivo studies are needed to evaluate the protective effect of CDDO-Me against cellular senescence in PTCs.

In conclusion, our results demonstrated a beneficial effect of CDDO-Me on cellular senescence in HK-2 cells. It is hypothesized that this beneficial effect is related to Nrf2 activation. CDDO-Me appears to be a candidate therapeutic for AKI. Our findings also showed that CDDO-Me induced apoptosis in cisplatin-treated HK-2 cells and potentially protects the kidneys from cellular senescence. Future studies are needed to assess the effects of CDDO-Me-induced apoptosis on cellular senescence and kidney function in AKI animal models.

## Supplementary Information

Below is the link to the electronic supplementary material.

Supplementary file1 (DOCX 22 kb)

## Data Availability

The data used to support the findings of this study are included within the article. All data generated or analyzed during this study are included in this published article and its Supplementary Information.

## References

[1] Chawla LS, Eggers PW, Star RA, Kimmel PL (2014). Acute kidney injury and chronic kidney disease as interconnected syndromes. N Engl J Med.

[2] Coca SG, Singanamala S, Parikh CR (2012). Chronic kidney disease after acute kidney injury: a systematic review and meta-analysis. Kidney Int.

[3] James MT, Hemmelgarn BR, Wiebe N, Pannu N, Manns BJ, Klarenbach SW (2010). Glomerular filtration rate, proteinuria, and the incidence and consequences of acute kidney injury: a cohort study. Lancet.

[4] Takaori K, Nakamura J, Yamamoto S, Nakata H, Sato Y, Takase M (2016). Severity and frequency of proximal tubule injury determines renal prognosis. J Am Soc Nephrol.

[5] Westhoff JH, Hilgers KF, Steinbach MP, Hartner A, Klanke B, Amann K (2008). Hypertension induces somatic cellular senescence in rats and humans by induction of cell cycle inhibitor p16INK4a. Hypertension.

[6] Satriano J, Mansoury H, Deng A, Sharma K, Vallon V, Blantz RC (2010). Transition of kidney tubule cells to a senescent phenotype in early experimental diabetes. Am J Physiol Cell Physiol.

[7] de Magalhães JP, Passos JF (2018). Stress, cell senescence and organismal ageing. Mech Ageing Dev.

[8] Childs BG, Durik M, Baker DJ, van Deursen JM (2015). Cellular senescence in aging and age-related disease: from mechanisms to therapy. Nat Med.

[9] Campisi J, d'Adda di Fagagna F (2007). Cellular senescence: when bad things happen to good cells. Nat Rev Mol Cell Biol.

[10] Soto-Gamez A, Demaria M (2017). Therapeutic interventions for aging: the case of cellular senescence. Drug Discov Today.

[11] van Deursen JM (2014). The role of senescent cells in ageing. Nature.

[12] Zhu Y, Tchkonia T, Pirtskhalava T, Gower AC, Ding H, Giorgadze N (2015). The Achilles’ heel of senescent cells: from transcriptome to senolytic drugs. Aging Cell.

[13] Wang E (1995). Senescent human fibroblasts resist programmed cell death, and failure to suppress bcl2 is involved. Cancer Res.

[14] Fuhrmann-Stroissnigg H, Ling YY, Zhao J, McGowan SJ, Zhu Y, Brooks RW (2017). Identification of HSP90 inhibitors as a novel class of senolytics. Nat Commun.

[15] Kim YM, Seo YH, Park CB, Yoon SH, Yoon G (2010). Roles of GSK3 in metabolic shift toward abnormal anabolism in cell senescence. Ann NY Acad Sci.

[16] Baar MP, Brandt RMC, Putavet DA, Klein JDD, Derks KWJ, Bourgeois BRM (2017). Targeted apoptosis of senescent cells restores tissue homeostasis in response to chemotoxicity and aging. Cell.

[17] Chang J, Yingying W, Shao L, Laberge RM, Demaria M, Campisi J (2016). Clearance of senescent cells by ABT263 rejuvenates aged hematopoietic stem cells in mice. Nat Med.

[18] Yosef R, Pilpel N, Tokarsky-Amiel R, Biran A, Ovadya Y, Cohen S (2016). Directed elimination of senescent cells by inhibition of BCL-W and BCL-XL. Nat Commun.

[19] Liby K, Hock T, Yore MM, Suh N, Place AE, Risingsong R (2005). The synthetic triterpenoids, CDDO and CDDO-imidazolide, are potent inducers of heme oxygenase-1 and Nrf2/ARE signaling. Cancer Res.

[20] Dinkova-Kostova AT, Liby KT, Stephenson KK, Holtzclaw WD, Gao X, Suh N (2005). Extremely potent triterpenoid inducers of the phase 2 response: correlations of protection against oxidant and inflammatory stress. Proc Natl Acad Sci USA.

[21] Yates MS, Tauchi M, Katsuoka F, Flanders KC, Liby KT, Honda T (2007). Pharmacodynamic characterization of chemopreventive triterpenoids as exceptionally potent inducers of Nrf2-regulated genes. Mol Cancer Ther.

[22] Sporn MB, Liby KT, Yore MM, Fu L, Lopchuk JM, Gribble GW (2011). New synthetic triterpenoids: potent agents for prevention and treatment of tissue injury caused by inflammatory and oxidative stress. J Nat Prod.

[23] Ruiz S, Pergola PE, Zager RA, Vaziri ND (2013). Targeting the transcription factor Nrf2 to ameliorate oxidative stress and inflammation in chronic kidney disease. Kidney Int.

[24] Pergola PE, Raskin P, Toto RD, Meyer CJ, Huff JW, Grossman EB (2011). Bardoxolone methyl and kidney function in CKD with type 2 diabetes. N Engl J Med.

[25] de Zeeuw D, Akizawa T, Audhya P, Bakris GL, Chin M, Christ-Schmidt H (2013). Bardoxolone methyl in type 2 diabetes and stage 4 chronic kidney disease. N Engl J Med.

[26] Chin M, Lee CY, Chuang JC, Bumeister R, Wigley WC, Sonis ST (2013). Bardoxolone methyl analogs RTA 405 and dh404 are well tolerated and exhibit efficacy in rodent models of Type 2 diabetes and obesity. Am J Physiol Ren Physiol.

[27] Ding Y, Stidham RD, Bumeister R, Trevino I, Winters A, Sprouse M (2013). The synthetic triterpenoid, RTA 405, increases the glomerular filtration rate and reduces angiotensin II-induced contraction of glomerular mesangial cells. Kidney Int.

[28] Wang YY, Yang YX, Zhao R, Pan ST, Zhe H, He ZX (2015). Bardoxolone methyl induces apoptosis and autophagy and inhibits epithelial-to-mesenchymal transition and stemness in esophageal squamous cancer cells. Drug Des Dev Ther.

[29] Shord SS, Thompson DM, Krempl GA, Hanigan MH (2006). Effect of concurrent medications on cisplatin-induced nephrotoxicity in patients with head and neck cancer. Anticancer Drugs.

[30] Yu W, Chen Y, Dubrulle J, Stossi F, Putluri V, Sreekumar A (2018). Cisplatin generates oxidative stress which is accompanied by rapid shifts in central carbon metabolism. Sci Rep.

[31] Sun X, Shi B, Zheng H, Min L, Yang J, Li X (2018). Senescence-associated secretory factors induced by cisplatin in melanoma cells promote non-senescent melanoma cell growth through activation of the ERK1/2-RSK1 pathway. Cell Death Dis.

[32] Li C, Xie N, Li Y, Liu C, Hou FF, Wang J (2019). N-acetylcysteine ameliorates cisplatin-induced renal senescence and renal interstitial fibrosis through sirtuin1 activation and p53 deacetylation. Free Radic Biol Med.

[33] Li W, Wang W, Li Y, Wang W, Wang T, Li L (2014). Proteomics analysis of normal and senescent NG108-15 cells: GRP78 plays a negative role in cisplatin-induced senescence in the NG108-15 cell line. PLoS ONE.

[34] Kurosaki Y, Imoto A, Kawakami F, Yokoba M, Takenaka T, Ichikawa T (2018). Oxidative stress increases megalin expression in the renal proximal tubules during the normoalbuminuric stage of diabetes mellitus. Am J Physiol Ren Physiol.

[35] de Zeeuw D, Akizawa T, Agarwal R, Audhya P, Bakris GL, Chin M (2013). Rationale and trial design of Bardoxolone Methyl Evaluation in Patients with Chronic Kidney Disease and Type 2 Diabetes: the Occurrence of Renal Events (BEACON). Am J Nephrol.

[36] Okada A, Nangaku M, Jao TM, Maekawa H, Ishimono Y, Kawakami T (2017). d-serine, a novel uremic toxin, induces senescence in human renal tubular cells via GCN2 activation. Sci Rep.

[37] Shimizu H, Bolati D, Adijang A, Muteliefu G, Enomoto A, Nishijima F (2011). NF-κB plays an important role in indoxyl sulfate-induced cellular senescence, fibrotic gene expression, and inhibition of proliferation in proximal tubular cells. Am J Physiol Cell Physiol.

[38] Jia Y, Kang X, Tan L, Ren Y, Qu L, Tang J (2021). Nicotinamide mononucleotide attenuates renal interstitial fibrosis after AKI by suppressing tubular DNA damage and senescence. Front Physiol.

[39] Samikkannu T, Thomas JJ, Bhat GJ, Wittman V, Thekkumkara TJ (2006). Acute effect of high glucose on long-term cell growth: a role for transient glucose increase in proximal tubule cell injury. Am J Physiol Ren Physiol.

[40] Gire V, Dulic V (2015). Senescence from G2 arrest, revisited. Cell Cycle.

[41] Donaldson KL, Goolsby GL, Wahl AF (1994). Cytotoxicity of the anticancer agents cisplatin and taxol during cell proliferation and the cell cycle. Int J Cancer.

[42] Itoh K, Chiba T, Takahashi S, Ishii T, Igarashi K, Oyake T (1997). An Nrf2/small Maf heterodimer mediates the induction of phase II detoxifying enzyme genes through antioxidant response elements. Biochem Biophys Res Commun.

[43] Aleksunes LM, Goedken MJ, Rockwell CE, Thomale J, Manautou JE, Klaassen CD (2010). Transcriptional regulation of renal cytoprotective genes by Nrf2 and its potential use as a therapeutic target to mitigate cisplatin-induced nephrotoxicity. J Pharmacol Exp Ther.

[44] Atilano-Roque A, Wen X, Aleksunes LM, Joy MS (2016). Nrf2 activators as potential modulators of injury in human kidney cells. Toxicol Rep.

[45] Plesca D, Mazumder S, Almasan A (2008). DNA damage response and apoptosis. Methods Enzymol.

[46] Islam MA, Sooro MA, Zhang P (2018). Autophagic regulation of p62 is critical for cancer therapy. Int J Mol Sci.

[47] Jung KT, Oh SH (2019). Polyubiquitination of p62/SQSTM1 is a prerequisite for Fas/CD95 aggregation to promote caspase-dependent apoptosis in cadmium-exposed mouse monocyte RAW264.7 cells. Sci Rep.

[48] Gunness P, Aleksa K, Kosuge K, Ito S, Koren G (2010). Comparison of the novel HK-2 human renal proximal tubular cell line with the standard LLC-PK1 cell line in studying drug-induced nephrotoxicity. Can J Physiol Pharmacol.

[49] Paolicchi A, Sotiropuolou M, Perego P, Daubeuf S, Visvikis A, Lorenzini E (2003). Gamma-glutamyl transpeptidase catalyses the extracellular detoxification of cisplatin in a human cell line derived from the proximal convoluted tubule of the kidney. Eur J Cancer.

[50] Jankinson SE, Chung GW, Loon EV, Bakar NS, Daizell AM, Brown CDA (2012). The limitations of renal epithelial cell line HK-2 as a model of drug transporter expression and function in the proximal tubule. Pflugers Arch.

