# Supplementary material for: In vitro study on effect of bardoxolone methyl on cisplatin-induced cellular senescence in human proximal tubular cells
Source: Mol Cell Biochem. 2022 Jan 1;477(3):689–99. doi: 10.1007/s11010-021-04295-y (PMC8857011; doi:10.1007/s11010-021-04295-y)
Supplement: Supplementary file 1 — Supplementary file1 (DOCX 22 kb) [file 11010_2021_4295_MOESM1_ESM.docx]

Supplement Table 1 Primer sequences for qPCR

| Gene | Primer sequence |
| --- | --- |
| *CDKN2A* | Forward 5′-TCCCTCAGACATCCCCGATT-3′ |
|  | Reverse 5′-GTGAAAAGGCAGAAGCGGTG-3′ |
| *CDKN1A* | Forward 5′-TCCTCATCCCGTGTTCTCCT-3 |
|  | Reverse 5′-CACCCTGCCCAACCTTAGAG-3′ |
| *CCND1* | Forward 5′-GGCGGAGGAGAACAAACAGA-3′ |
|  | Reverse 5′-TGTGAGGCGGTAGTAGGACA-3′ |
| *MKI67* | Forward 5′-CGTCCCAGTGGAAGAGTTGT-3′ |
|  | Reverse 5′-CCTTCCAAACAAGCAGGTGC-3′ |
| *IL6* | Forward 5′-AGTGAGGAACAAGCCAGAGC-3′ |
|  | Reverse 5′-AGCTGCGCAGAATGAGATGA-3′ |
| *IL8* | Forward 5′-TCTGCAGCTCTGTGTGAAGG-3′ |
|  | Reverse 5′-TTCCTTGGGGTCCAGACAGA-3′ |
| *HO1* | Forward 5′-GCCCTTCAGCATCCTCAGTT-3’ |
|  | Reverse 5′-CACGCATGGCTCAAAAACCA-3′ |
| *NQO1* | Forward 5′-GAAAGGATGGGAGGTGGTGG-3′ |
|  | Reverse 5′-TGGCAGCGTAAGTGTAAGCA-3′ |
| *SOD1* | Forward 5′-GACTGACTGAAGGCCTGCAT-3′ |
|  | Reverse 5′-ATCGGCCACACCATCTTTGT-3′ |
| *GPX1* | Forward 5′-TTCGAGCCCAACTTCATGCT-3′ |
|  | Reverse 5′-CGATGTCAGGCTCGATGTCA-3′ |
| *CAT* | Forward 5′-AGTGATCGGGGGATTCCAGA-3′ |
|  | Reverse 5′-GAGGGGTACTTTCCTGTGGC-3′ |
| *GAPDH* | Forward 5′-TGGCCTTCCGTGTTCCTACCC-3′ |
|  | Reverse 5′-CCGCCTGCTTCACCACCTTCT-3′ |
